# Social markers in the care of intrafamilial child sexual abuse: A socio‐representational approach and its theoretical and methodological implications

**DOI:** 10.1111/bjso.70145

**Published:** 2026-10-22

**Authors:** Ophélie Gallardo, Thémis Apostolidis, Solveig Lelaurain

**Affiliations:** ^1^ Aix Marseille Univ, LPS Aix‐en‐Provence France

**Keywords:** care, child sexual abuse, incest, professional judgement, professional practice, psychosocial approach, social markers, social representations

## Abstract

Professionals responding to intrafamilial child sexual abuse (CSA) must assess ambiguous situations shaped by emotional tension and social knowledge. This research investigates the socio‐cognitive logic guiding their judgements and intended practices in the French context, where legal and institutional reforms have strengthened expectations for identification and protection of CSA. It draws on social representations theory to shed light on how responses are shaped by socio‐symbolic meanings. A two‐step methodology was implemented. Study 1 developed a contextualised vignette reflecting the ambiguity in intrafamilial CSA disclosures. Thirty interviews with the general population were analysed to identify interpretative frameworks activated in evaluation, informing vignette operationalisation and assessment measures. Study 2 used this material to examine the influence of victims' social markers (age: 8 vs. 14 years; socioeconomic background: advantaged vs. disadvantaged) on judgements among 380 professionals‐in‐training in healthcare and social work. Results showed that these social markers affected severity assessments, responsibility attribution to the minor and alleged perpetrator and decisions regarding protective interventions. Findings highlight socio‐representational logic related to age and social class that orient interpretation and decision‐making. This work underscores the need for reflexive training to reduce influences in CSA responses and demonstrates the value of contextualised methodological approaches for advancing research and practice.

## INTRODUCTION

### Care practices as social practices: The case of sexual violence against minors

The care of intrafamilial child sexual abuse (CSA) takes place within a social environment shaped by norms, values and ideologies. Professionals are not neutral: they are both products and producers of this sociocultural context. Their work takes place at the intersection of multiple interactions – with victims, families, and peers – embedded in and contributing to the broader social context (Clark et al., 1991). From this psychosocial perspective, care practices cannot be reduced to technical gestures or individual responses; they are social practices, situated and imbued with meaning, reflecting shared systems of thought (Morin & Apostolidis, 2002; Twigg & Byrne, 2014). Professional practices are regulated by lived experiences, professional and social knowledge, interactions, affective and socio‐symbolic links, socio‐cognitive determinants, social positions, and power relations (Oswald, 2013) – for example, sexism, adultism, racism and classism. They therefore carry social meaning (Abric, 1994; Jodelet, 1989), which becomes particularly salient when professionals are confronted with certain situations.

A recent scoping review (Gallardo et al., 2026) highlighted the fact that these professional judgements are shaped by socio‐cognitive determinants, particularly the social markers attributed to victims. It also revealed cultural variations, suggesting that these mechanisms are embedded in broader social structures rather than arising solely from individual attitudes. Social markers refer to socially constructed attributes (e.g., age, gender or social class). Situations of violence, the people involved and care are interpreted through these attributes, guiding perceptions, judgements and responses. These attributes activate categorisation processes (Kang & Bodenhausen, 2015; Tajfel, 1981) that influence how victims' accounts and behaviours are received (Davies & Rogers, 2009). According to Doise (1993), social marking operates at the intersection of social regulations (e.g., norms, status) and cognitive processes (e.g., categorisation and judgement) within a specific situation – that is, social dimensions influence how individuals think.

Age, understood as a social construct, illustrates this dynamic. It organises individuals into life stages – here, childhood and adolescence – associated with common‐sense assumptions about sexuality, innocence, credibility, vulnerability, risk‐taking and responsibility (Bonnet, 2010; Brey et al., 2022). These beliefs can lead professionals to assign greater responsibility to adolescents than to children when assessing sexual violence (Aleissa et al., 2022; Wangamati et al., 2020), limiting recognition and reporting of abuse (Gallardo et al., 2026). Similarly, social background is another marker subject to socio‐economic and racial prejudices that can render some at‐risk minors invisible or produce guilt‐inducing responses toward them (Otterman et al., 2024). Thus, the care of CSA must be understood as a social arena in which perceptions, attitudes, and interventions reflect the social structures surrounding both professionals and victims. This underscores the need for a contextualised and interpretive approach to fully grasp the logic involved.

### Socio‐representational approach to a complex and sensitive field of care

The social representations theory (SRT; Moscovici, 1984; Sammut et al., 2015) offers a heuristic conceptual framework for applying a psychosocial perspective to the care of CSA. Social representations (SR) are ‘a form of knowledge, socially constructed and shared, with a practical purpose and contributing to the construction of a common reality for a social group’ (Jodelet, 1989, p. 36). In the present study, we adopt an integrative approach to the SRT, connecting complementary perspectives on SR in order to jointly examine representational content, social processes, social practices, and the socio‐ideological contexts in which they are produced and brought into play (Apostolidis, 2006). Following Jodelet's sociogenetic approach, SR are understood as socially situated forms of knowledge that emerge through interactions, practices and socio‐historical contexts. They inform both how individuals relate to a phenomenon and how this relationship is embedded within a given social order (Jodelet, 2015). They are shaped by the individual (history and experiences), the social and ideological system in which they are embedded, and their connections with this social system (Abric, 2003). SR are thus understood as socio‐cognitive constructions involving both individual and collective dimensions (Oswald, 2013). From this perspective, drawing on the SRT enables the examination of the system of values, norms and social relations in which these representations are embedded, which may vary across individuals and social contexts. Analysing care provision (e.g., decisions, attitudes and practices) through the SRT therefore means considering it as a symbolic social practice. CSA situations activate and reveal judgements, attitudes and professional practices embedded in social logic. Complementing the sociogenetic perspective, Doise's societal approach (Doise, 1993, 2003, 2011) emphasises how social positions, norms and ideological systems shape representational processes and social judgements. As Doise (2003) notes, ‘judgements are, at the individual level, instances through which attitudes actualize social relations and are organized into social representations’ (p. 256). The sociodynamic approach thus connects the cognitive system and the social metasystem (Doise, 2011), taking into account the social structure underlying communication and interpersonal relations (Apostolidis, 2006). SR must therefore be understood as both cognitive content and social processes (Moscovici, 2008), making it possible to study the way in which professional judgements mediate social logic.

The SRT provides a framework for analysing the social and symbolic logic underlying professional reasoning, judgements, attitudes and practices towards a phenomenon, linking representational content, social functions and the context of action. It therefore offers an interpretative framework through which to examine the social, ethical, medical, relational and legal issues of care practices. In this regard, it is essential to consider the functions of SR (Abric, 1994; Jodelet, 1989). Understanding their functions helps reveal how protagonists make sense of, interpret and position themselves within the multiple issues involved in care provision. An initial knowledge function supports understanding and communication. It provides meaning to everyday events by enabling interpretation based on pre‐existing knowledge, while also contributing to the development of common sense by circulating and transforming knowledge (Oswald, 2013). A second orientation function guides behaviours and practices: SR serve as a ‘guide for action or inaction’ (Abric, 1994, p. 13) towards victims, perpetrators, and third parties (e.g., relatives, professionals and society), shaping responses to social reality (Abric, 2003). A justificatory function legitimises positions and behaviours a posteriori. Finally, an identity function helps define group identity and specificity (Abric, 1994; Jodelet, 1989). These functions contribute to the construction of shared meaning around a phenomenon experienced as emotionally sensitive, morally charged and socially conflictual.

The SRT therefore also provides tools for interpreting, appropriating, translating and regulating social and emotional uncertainty by making the unknown familiar (Moscovici, 1976). Theorising ambiguity through the SRT implies acknowledging that care provision is not solely governed by technical or legal rationales but is embedded in complex affective, relational and socio‐symbolic dynamics. The SRT enables the contextualisation of discourses and the identification of how SR systems are mobilised – sometimes in contradictory ways – when individuals face uncertainty. As Abric (2016) emphasises, ‘representations play an increasingly important role when actors are confronted with complex or ambiguous situations’ (p. 283). Situations characterised by multiple interpretative registers (Moscovici, 1984), in which socio‐affective dimensions are salient and professional knowledge is insufficient (Restivo et al., 2016), are particularly permeable to common‐sense beliefs. They provide interpretative frameworks aligned with dominant social representations and norms. By making the unthinkable thinkable and the unspeakable speakable, the SRT sheds light on how practices are built not only on knowledge but also on situated social and symbolic relations. As such, SR are more than ways of thinking: they are ways of acting and (re)constructing reality (Moscovici, 1984; Oswald, 2013), notably in contexts where ethics, knowledge, emotions and norms are closely intertwined.

### The French context as a revelatory case for studying socio‐representational dynamics in CSA care

The French context is a particularly revelatory environment for examining the socio‐representational dynamics underlying professional responses to CSA. Over the past decade, institutional, legal, and societal transformations have reshaped expectations regarding the recognition and care of CSA, while uncertainty and variability in professional practices persist. This coexistence of normative change and practical resistance provides a particularly fertile context for studying how SR guide professional judgements and practices.

In recent years, several publicised cases of CSA and incest, including testimonies by Adèle Haenel and Camille Kouchner, have transformed public debate in France. These disclosures have challenged the invisibility of sexual violence against children and contributed to the questioning of institutional responses, professional responsibilities and dominant beliefs surrounding it. This societal mobilisation has been accompanied by institutional and legislative reforms, such as the Haute Autorité de Santé's^1^ recommendations for systematic screening (Haute Autorité de Santé, 2019), the creation in 2021 of the Independent Commission on Incest and Sexual Violence Against Children (CIIVISE) tasked with promoting a culture of prevention and protection (CIIVISE, 2023). Other developments include the 2021 Billon law protecting minors from sexual crimes and incest (French Republic, 2021), the national plan to combat violence against children (2023–2027) and recent legislative changes, notably to Article 223–14 of Law n°2024–420, allowing exceptions to professional secrecy regarding CSA (French Republic, 2024). These developments redefine expectations for healthcare professionals (e.g., physicians, nurses and psychologists) and those in the socio‐educational sector (e.g., social workers, youth workers and support staff). They are positioned as key actors in primary (i.e., screening and detection), secondary (i.e., support for victims) and tertiary prevention (i.e., raising awareness among the public) (Coinçon, 2019; Haute Autorité de Santé, 2011; Hatem‐Gantzer, 2021).

However, these transformations have not eliminated uncertainty in professional practice (Gallardo et al., 2026). Despite this regulatory framework, significant knowledge gaps remain (Otterman et al., 2024), contributing to difficulties in interpreting and responding to CSA situations. These challenges are amplified by the difficulty of delineating sexual violence, which can take many forms and result in diverse experiences and consequences. Some situations perceived as ‘ambiguous’ (Gallardo et al., 2026; Hovey et al., 2013) raise questions among professionals regarding their qualification and severity, as they do not align with dominant social norms. To this factual ambiguity is added that of disclosure: first‐time revelations are not always clearly or precisely articulated by victims. They may be fragmented, minimised or trivialised. Memory may be impaired or traumatised; the words needed to describe the abuse may be lacking or unknown. Faced with these grey areas, professionals draw on common‐sense knowledge to guide judgement and decision‐making (Restivo et al., 2016). Myths and stereotypes surrounding CSA contribute to resistance in recognising and responding appropriately to such violence (Gallardo et al., 2026). In France, 6 out of 10 professionals report having been unable to protect a minor following a disclosure (CIIVISE, 2023). Only 15% of victims reported disclosing their abuse for the first time to a healthcare professional (CIIVISE, 2023). This low level of recourse to protection and care institutions may reflect the trivialisation of abuse severity, or a lack of confidence in institutions perceived as neither supportive nor protective (CIIVISE, 2023).

Despite recent social and legal advances, the care of CSA remains shaped by the complexity of cases and by persistent beliefs that contribute to obscuring, minimising, or even legitimising violence (Romito, 2006), thereby increasing the risk of secondary victimisation. From a socio‐representational perspective, the coexistence of evolving institutional norms and socio‐representational beliefs is particularly informative because periods of normative transformation create situations in which established professional routines are challenged while new norms remain only partially stabilised. In such contexts, professionals interpret uncertain situations by drawing on institutional guidance but also on socially shared knowledge, beliefs, stereotypes and values. Examining professional judgements within this context therefore provides insight into the socio‐representational processes through which evolving social norms are appropriated, negotiated or resisted in everyday practice.

In a context where professional practices are strained by institutional tensions, uncertainty, and resistance, the SRT functions as a heuristic tool for making visible the social logic structuring discourse and professional practices, thereby supporting a better understanding of the obstacles to effective, ethical, and protective intervention.

## CURRENT RESEARCH

This research aims to analyse the socio‐cognitive logic that shapes professional judgements and practices regarding intrafamilial CSA through a two‐part complementary design.

Study 1 focuses on the development of contextualised and ambiguous material. The first objective is thus to describe the methodological approach used to construct anchored material based on an analysis of intrafamilial CSA, designed to capture judgements in a professional intervention context. This section details this qualitative procedure in order to highlight the importance of contextualised operationalisation through the use of a scripted vignette. The invisibilisation and strong moral condemnation that characterise intrafamilial CSA make it a complex subject of study. The pervasive ambiguity in disclosure situations serves here as a means of accessing the representations drawn on.

Study 2 makes use of the material developed in Study 1 to examine the socio‐cognitive logic drawn on by future professionals when responding to an ambiguous situation of intrafamilial CSA. The second objective was to examine whether variations in the victim's social markers, namely age and socioeconomic background, were associated with differences in professional judgements and intended practices.

### Ethical statements

Both studies received approval from the Ethics Committee [AixMarseilleUniv; 2024‐05‐23‐01]. All participants provided informed consent prior to participation. In Study 1, participants were informed about the objectives and procedures of the interview study before providing consent. In Study 2, participants were informed of the research procedure and received an experimental debriefing after participation, in accordance with the ethical requirements for informed consent as defined in the Psychologists' Code of Ethics. This debriefing aimed, first, to remind participants of the research objectives and inform them of the fictitious nature of the scenario; and second, to provide support resources to individuals who might feel the need for assistance. Contact information for specialised organisations offering free, anonymous, and supportive listening services was provided.

## STUDY 1: METHODOLOGICAL DEVELOPMENT OF CONTEXTUALISED MATERIAL

Study 1 aimed to highlight the contextualised and qualitative operationalisation for designing the experimental material used in Study 2.

### Construction of the vignette: Sociological anchoring and manipulated variables

To address the issue of professional intervention, a scripted vignette method was selected. This tool introduces a situation embedded in a socially meaningful context, activating the associated social knowledge. To ensure realism and authenticity, the vignette was constructed using statistical data: in 80% of cases, CSA occurs within the family, is committed by a male from an older generation, and mainly concerns girls (CIIVISE, 2023). These elements were standardised across vignettes to ensure ecological validity (Deconchy, 2002; Harrits & Møller, 2020), reflecting a sociocultural context marked by adultism and patriarchy. The construction also drew on victim accounts and professional experience to strengthen plausibility.

The scripted vignette method also allows for controlled variation of information in order to examine the effect of specific variables (Harrits & Møller, 2020) and the operationalisation of ‘envelope situations’ of complex social processes (Deconchy, 2002), that is, an arrangement of social stimuli designed to create a socially consistent situation in which cognitive and ideological processes can be elicited. Based on these considerations, two dimensions were manipulated: the victim's age and her social background. Regarding age, the minor was described either as 8 years old, referring to childhood, or 14 years old, referring to adolescence. Concerning social background, this was induced through parents' occupational categories: an unemployed father and a housekeeper mother for a disadvantaged social background, and a notary father and a commercial manager mother for an advantaged social background.

Once these elements were fixed, the objective was to observe variations in perceptions depending on the clarity of the reported facts. Two versions of the vignette were therefore created: one presenting sexual violence explicitly, and the other implicitly. In the explicit version, sexual touching was described in detail, facilitating recognition of the violence. In contrast, in the implicit version, the minor's feelings were emphasised, enabling exploration of participants' reactions to a narrative in which the violence was concealed and uncertain. To identify socio‐cognitive processes in context, participants were asked to imagine themselves in an intervention scenario. The vignette was thus intended to trigger interpretative analyses.

Finally, specific attention was paid to the narrative form (Harrits & Møller, 2020). The vignette had to be adapted for both 8 year old and 14 year old minors, requiring careful consideration of the narrative mode and vocabulary choice to avoid overly technical terms.

### Participants and procedure: Evaluation of the vignettes

To test the different versions of the vignettes, 30 semi‐structured interviews of approximately 50 min were conducted (by author 1: Gallardo). Participants were members of the general population (M_age = 32 years), predominantly women (*n* = 18). Fourteen participants were students enrolled in a range of academic programs, including psychology (*n* = 8), while the remaining participants came from diverse occupational backgrounds. They were recruited via social media and snowball sampling and calls for participation were posted on the researchers' professional social media accounts (LinkedIn and Facebook). The aim was to progressively refine the tool: the explicit version and the first implicit version of the vignette were used during the first 16 interviews, while the refined version was used in the remaining 14 interviews. An iterative, theory‐driven process using researcher triangulation (Restivo et al., 2023), combining theoretical, methodological and empirical insights, enabled progressive adjustments to the material throughout data collection until a final version was obtained (see Text 1). Despite these successive refinements, all versions retained the same narrative framework: a minor disclosing to a school nurse an event perceived as uncomfortable, involving her father. During the interviews, participants were first invited to freely describe the situation presented in the vignette. Follow‐up questions explored their perceptions of the victim, the alleged perpetrator, the family context, the school nurse's probable response, the severity and nature of the situation and the realism of the scenario. The complete interview guide is provided in Appendix S1.

TEXT 1 **Final vignette**

‘At the end of the morning, Clara is called in by the school nurse for an annual health check. Clara, aged 8/14, is in 2nd grade/9th grade at a school/middle school in Marseille. She lives with her two parents and her younger brother. Her father is unemployed/a notary, her mother works as a housekeeper/a commercial manager, and her brother is in the last year of preschool.

During the consultation, the nurse asks her various questions. Clara then begins to speak about a situation that happened a few weeks ago. She explains to the nurse that when her father came home/from the office, he went into her room to say goodnight. She says that, as usual, her father hugged her, but for her, these hugs felt ‘weird’. Clara reports feeling uncomfortable, but she did not say anything to her father’.

The conversation between Clara and the nurse continued.

### Analysis

The interview data were analysed through a targeted qualitative content analysis using a deductive and iterative coding process (Elo & Kyngäs, 2008), aimed at refining the vignette rather than producing a comprehensive qualitative account. The analysis focused on predefined aspects of the vignette (e.g., spontaneous identification of abuse, perceived severity, representations of the minor, the father and the family), while categories were iteratively refined and related throughout the analytic process. The first author systematically compared participants' comments in order to identify recurring interpretations, points of convergence and divergence, and suggestions regarding the plausibility, ambiguity and wording of the scenario. These observations were discussed with the research team after successive interviews, leading to revisions of both the coding grid and the vignette until a satisfactory version was obtained.

### Results of the interviews

The analysis revealed that both the ambiguous and explicit versions of the vignette triggered similar reactions among participants. As illustrated in the excerpts below, participants quickly identified the presence of abuse, leading to strong aversive reactions and calls for sanctioning the father. This polarisation appeared to stem from the incestuous climate conveyed in both versions of the vignette, notably through expressions such as ‘sharing a secret’, ‘absent mother’, ‘alone with her father’, ‘caress’, and ‘touch’. Accordingly, the analysis of participants' responses showed that once they had read the full vignette, they expressed no doubt regarding the incestuous nature of the situation, whether described explicitly or implicitly.

*For me, it's **sexual abuse**, the fact that the father is touching her… If I had stopped here [points halfway through the vignette], she could have thought that and it could have been just her own thoughts. But after reading everything, it's real **sexual abuse** for me* (Leslie, 23).^2^

*While reading, I suspected **something like this** was going to happen. I don't know if it's the writing style or because we hear a lot about **these things** at the moment* (Andréa, 28).

This type of narration, by depicting a salient environment of abuse, activated a pathologising representation of the perpetrator as inherently deviant. The father was thus immediately described as a ‘*mentally ill person*’, ‘*pervert*’, ‘*monster*’ or ‘*abnormal*’. Participants' discourse was marked by strong moral condemnation, as seen in the vocabulary used to describe the perpetrator. He was judged to be outside the realm of normality, no longer perceived as a ‘*person like any other*’. As a result, sexual abuse was relegated to the domain of exception, making it more difficult to recognise perpetrators embedded within ordinary or familial contexts. Thus, the writing and narrative environment appeared to saturate participants' interpretations of the father and the situation, regardless of the manipulated independent variables.

***Sick**, you can't be anything other than **seriously sick**, there's **something wrong** with you…* (Loïc, 54).

*I see the father as **sick, disturbed, perverted, disgusting**. […] **You can't be normal** to do that. Because if you're normal, you aren't **sexually attracted** to your children and if you do have an impulse, you control it* (Lucie, 50).

Following these initial observations, the explicit version of the vignette was removed, and the implicit version was revised, particularly by eliminating vocabulary that created an incestuous climate, which was polarising participants' discourse. This revision led to more nuanced viewpoints. The implicit nature of the situation resulted in greater uncertainty in participants' responses, marked by conditional forms, expressions such as ‘*maybe*’ and ‘*we don't know*’ and the coexistence of multiple possible interpretations, including or excluding the recognition of sexual violence.

*She says her father **maybe** touched her or **maybe not. We don't know**… **There isn't enough detail**… She says she has the impression it was more than it should have been* (Franck, 55).

*What it made me think of is touching, **maybe more**, in any case sexual violence. But then I took a step back and thought: ‘She's in 2nd grade, does she really understand what happened? **Did something really happen?’*** (Marie, 23).

This ambiguity left room for interpretation, leading participants to draw on personal experiences and social knowledge to make sense of the situation. As illustrated by Manon's excerpt, lived experience enabled some behaviours to be normalised, contributing to differentiated representations based on intimate family norms.

***It's weird, but it could also be nothing**. When I was a kid, even until I was 15 or 16, I would kiss my father on the mouth and it was **just a relationship** between a girl and her dad* (Manon, 36).

By contrast, Mathilde drew on common representations of adolescence – as a period of identity‐related turbulence, attention‐seeking, bodily changes, and ambivalent attitudes towards nudity – to interpret the situation. In other words, social factors entered the analysis and participated in the construction of the thresholds for recognising violence.

***It could be so many things**… What's certain is that she's uncomfortable, but **there could be many situations**. It might be a **call for help**, or **wanting more attention**… Her **body is changing**, and every family has different **norms around nudity*** (Mathilde, 50).

Similarly, Quentin drew on several beliefs in his reasoning. First, he viewed children as potentially unreliable, capable of misunderstanding or misinterpreting reality, which can undermine their credibility. Second, he appealed to the presumption of innocence by questioning the father's intent. The hypothesis of harmful intent was quickly counterbalanced by situational justifications such as fatigue or context. This reflects a socially shared belief that parents are presumed to be caring unless irrefutable proof to the contrary is presented and reveals a fear of wrongful accusation that can take precedence over acknowledgement of the child's distress. Finally, his focus on intent conveyed a representation of sexual abuse as necessarily involving deliberate harmful intent, thereby making its recognition contingent on establishing the perpetrator's intentions rather than on the nature of the acts and their impact on the child.

*To me **it's ambiguous**, because the child **may** **be interpreting** gestures, a word or **I don't know**, a kiss, **maybe** it triggered something in the child that isn't the reality. Then I think: does her father have bad **intentions**? Maybe also. And then I think: a father can hold his child in his arms, **he may have been** tired that day, **it may be** different from usual but still **paternal**. So yes, he acts differently because he's tired, had a bad day, it's different from usual but it doesn't necessarily have a… Well, we won't say perverse but a perverse connotation as the child feels it* (Quentin, 60).

### Final modifications to the vignette and conclusion

This qualitative vignette development process showed that narrative ambiguity – and consequently the implicit nature of the violence – creates space for participants' imagination. This openness acts as a marker of the interpretative frameworks they draw on, where social knowledge and lived experience structure their attempts at explanation, the judgements they express, and the decisions they consider (Restivo et al., 2016). In this regard, the implicit vignette allows us to understand how representations are activated in the absence of explicit cues, offering insight into the social logic at work when evaluating violence. Furthermore, selecting a vignette that implicitly evokes violence responds to an ecological concern in research, reflecting the real‐world context in which initial disclosures of violence are often confusing and ambiguous. Finally, this theoretical‐methodological approach made it possible to refine the vignette narrative while avoiding a salient incestuous environment. Modulating the narration – and therefore the perceived context – emerges as a lever for exploring the diversity of beliefs, ideologies, and interpretative schemas activated among participants (Deconchy, 2002).

Beyond their methodological contribution, the qualitative interviews also provided analytical insight into the content of the SR drawn on when participants interpreted an ambiguous situation of intrafamilial CSA. Whereas the explicit version of the vignette activated a consensual and polarised representation of the situation, characterised by the immediate recognition of incest, the pathologisation of the father, and strong moral condemnation, the refined implicit version revealed the representational systems participants relied upon to interpret the situation. By reducing explicit cues, the interviews made it possible to observe the thresholds at which violence was recognised and qualified. Faced with uncertainty, participants drew on common‐sense knowledge and lived experience, on representational systems related to childhood and adolescence, family relationships and normative family practices, parental intentions, children's credibility and the presumption of parental innocence to construct meaning. These constitute the socio‐representational content called on for recognising, qualifying, and judging situations of intrafamilial CSA under conditions of ambiguity (Abric, 2016; Moscovici, 1976).

### Development of questionnaire items

The interviews conducted during the vignette construction also contributed to the development of the content of the dependent variables, relying on ad hoc material drawn directly from participants' representations and discourse. The objective was to anchor the questionnaire in common‐sense knowledge, constructing the items from the ways of thinking and language observed in the interviews rather than from researchers' knowledge or the scientific literature. The socio‐representational approach therefore enables the identification of contextualised realities and social knowledge specific to the social environment depicted in the vignette, by accounting for the meanings individuals attribute to the situation (Linhares et al., 2022). Based on the interviews, 120 items were formulated and classified in a table according to the dependent variables they were intended to measure. An initial selection, supported by researcher triangulation, was made based on relevance, redundancy, and clarity, reducing the number of items from 120 to 60. Specifically, this process involved reviews by three researchers, who examined the items and discussed their categorisation and formulation. Disagreements were resolved through discussion, ensuring that the retained items reflected the meanings expressed in participants' discourse while remaining consistent with the objectives of the study.

### Participants and procedure: Evaluation of questionnaire items

A second series of interviews (*n* = 30), lasting approximately 30 min, was conducted to ensure clarity of the 60 selected items and consistency between these items and the vignette. Participants were members of the general public, primarily trainees in professional programs (*n* = 28), mostly women (*n* = 23), recruited via social networks and outreach within a French university. During the interview, participants first completed the questionnaire, then discussed it with the interviewer. To collect their feedback, the interviewer asked: ‘How well do you understand the questionnaire items?’, ‘To what extent do the questions seem consistent with the situation presented at the beginning?’, and ‘Which elements appear redundant to you in the questionnaire?’

### Results and final modifications of the questionnaire items

The interviews revealed an imbalance in the initial questionnaire: most items directed participants towards interpreting the situation as a case of sexual violence, at the expense of other possible interpretative frameworks (e.g., a misunderstanding, a lie, a bid for attention). To limit this inductive effect and preserve the interpretative plurality of the vignette, new items were added, offering alternative explanations. This approach aimed not to constrain participants' reading of the situation within a single frame, but to allow them to project their own representations into it.

In addition, certain items proved open to multiple interpretations. For instance, the item *‘Clara is an innocent young girl’* could refer either to innocence associated with childhood or to moral innocence implying the truthfulness of her account. This kind of ambiguity led to the reformulation or removal of some items.

Following this phase, a final reduction was conducted: the total number of items decreased from 60 to 40. Redundant items and those that did not vary significantly across the independent variables were eliminated. This refinement process enhanced the clarity, coherence, and sensitivity of the items relative to the vignette. The complete questionnaire is provided in Appendix S2.

Finally, the interviews revealed variations in participants' discourse according to the manipulated independent variables. These differences strengthened the relevance of the final set of items by demonstrating their capacity to activate differentiated belief systems. These initial analytical findings thus support both the validity of the hypotheses and the robustness of the methodological design.

## STUDY 2: THE EFFECT OF SOCIAL MARKERS ON FUTURE PROFESSIONALS' JUDGEMENTS: AN EXPERIMENTAL STUDY

### Method

Study 2 builds on the material finalised at the conclusion of Study 1. It focuses on analysing participants' judgements regarding the described situation, the father, the minor and the type of professional response envisaged.

#### Participants

This study was conducted with 380 future professionals enrolled in training programs in the South of France: 210 in 10 nursing training institutes (Instituts de Formation en Soins Infirmiers; IFSI) and 170 in three Regional Institutes of Social Work (Instituts Régionaux du Travail Social; IRTS). They were distributed across 3 years of training (125 first, 132 s, 103 third, and 20 classified as ‘other’) during the 2024–2025 academic year. The participants’ mean age was 29 years. Of these, 87.4% (*n* = 332) identified as women, 11.8% (*n* = 45) as men and 0.8% (*n* = 3) identified otherwise. Most had no children (*n* = 271, 71.3%), and 26.6% (*n* = 101) reported having been exposed to sexually inappropriate behaviours and/or attitudes during childhood. A majority (*n* = 256, 67.4%) felt informed about the phenomenon of CSA, yet only 25% (*n* = 95) stated they had received specific training on this topic.

Sample size was determined based on previous literature and a sensitivity analysis using G⁎Power (Version 3.1.9.7; Faul et al., 2007). This analysis indicated that 179 participants were needed to detect a medium effect (d = .25, power = .80, *α* = .05). Because two distinct populations were studied, the minimum was doubled to 358 participants.

#### Procedure and materials

Data collection was conducted online via the LimeSurvey platform using a self‐administered questionnaire. Participants were recruited via email through university mailing lists. After reading the information sheet and providing consent, they were randomly assigned to one of four experimental conditions following a 2 × 2 factorial design: age (8 vs. 14 years old) × social background (disadvantaged vs. advantaged). They read the corresponding vignette (see Text 1) and then performed a free‐association task in which they were instructed to spontaneously produce three words that first came to mind in response to the vignette. This free‐association technique was used to explore the socio‐representational content spontaneously activated by future professionals when interpreting the situation. This method provides access to the implicit and spontaneous meanings associated with a given stimulus (Aeschbach et al., 2025).

Participants then completed four successive sections of the questionnaire, responding to each item on a 7‐point Likert scale (1 = *strongly disagree* to 7 = *strongly agree*). The first section assessed perceptions of the situation (6 items; e.g., ‘In my view, the situation described by Clara is surely a misunderstanding’; *α* = .86). The second section evaluated perceptions of the minor (8 items; e.g., ‘Like others of her age, Clara is likely in a seductive phase’; *α* = .86) and of her father (8 items; e.g., ‘Clara's father's gestures are certainly without harmful intent’; *α* = .86). The third section addressed the perceived adequacy of the professional response (13 items; e.g., ‘The school nurse will reassure Clara by telling her that nothing is abnormal’; *α* = .62). The fourth and final section collected socio‐demographic information.

#### Data analysis

Words generated through the free‐association task were first manually lemmatised by the researchers and then subjected to a qualitative content analysis before being analysed using chi‐square tests in SPSS (30.0.0.0 (172)). The lemmatisation process consisted of grouping morphological variants (e.g., singular and plural forms and different grammatical forms), whereas semantically equivalent terms (e.g., discussion, talking, exchange and dialogue) were subsequently grouped into common semantic categories through qualitative content analysis (Dany, 2016; Restivo et al., in press). This process, discussed among the researchers to ensure consistency, reduced the corpus from 457 distinct lexical forms to 264 words after lemmatisation and finally to 106 semantic categories following content analysis.

Items were grouped based on semantic analysis and an exploratory factor analysis (EFA). The analysis was conducted using SPSS, employing Principal Component Analysis with Varimax rotation. The questionnaire consisted of 35 items; reverse‐worded items were recoded to ensure consistent scoring. Data adequacy was good (KMO = .89; Bartlett's χ^2^ (595) = 4781.31, *p* < .001). Nine factors were retained using Kaiser's criterion (eigenvalues >1), explaining 62.84% of total variance. The factors were labelled as follows: Minimisation of the situation's severity (*n* = 5; *α* = .71); Non‐recognition of the abusive nature of the situation and its impact (*n* = 2; *α* = .68); Exoneration of the father (*n* = 7; *α* = .82); Sexualisation of the father's intentions (*n* = 2; *α* = .50); Blame and disqualification of the minor (*n* = 5; *α* = .82); Attribution of silence and inaction to the minor (*n* = 2; *α* = .58); Institutional inaction (*n* = 6; *α* = .71); Family mediation and internal investigation (*n* = 4; *α* = .59); Perceived negative consequences of reporting (*n* = 2; *α* = .61) (see Table 1). The effects of age and social background were first examined using a two‐way multivariate General Linear Model (MANOVA). When multivariate effects were identified, tests of between‐subject effects were used to examine each dependent variable separately.

**TABLE 1:** Independent and dependent variables included in the experimental design.

Variables	Type	Operationalisation
Victim's age	Independent variable	8 vs. 14 years
Social background	Independent variable	Disadvantaged vs. advantaged
Minimisation of the situation's severity	Dependent variable	5 items, *α* = .71
Non‐recognition of the abusive nature of the situation and its impact	Dependent variable	2 items, *α* = .68
Exoneration of the father	Dependent variable	7 items, *α* = .82
Sexualisation of the father's intentions	Dependent variable	2 items, *α* = .50
Blame and disqualification of the minor	Dependent variable	5 items, *α* = .82
Attribution of silence and inaction to the minor	Dependent variable	2 items, *α* = .58
Institutional inaction	Dependent variable	6 items, *α* = .71
Family mediation and internal investigation	Dependent variable	4 items, *α* = .59
Perceived negative consequences of reporting	Dependent variable	2 items, *α* = .61

### Results

The free‐association results (see Table 2) show that the situation generally evoked the themes of incest (*n* = 109) and fondling (*n* = 73), which appeared as the most frequently evoked terms. Furthermore, certain terms were significantly more or less frequently mentioned depending on the experimental conditions, suggesting that these conditions activate distinct representational worlds. When the family's social background was described as advantaged, participants associated the situation with ‘discomfort’, ‘unease’, and ‘hugging’. In contrast, when the social background was described as disadvantaged, the associations shifted towards aggression and rape. Regarding age, references to family and consent emerged when the minor was described as 14 years old, whereas rape was the predominant association when the child was described as 8 years old.

**TABLE 2:** Frequencies of lexical items associated with the situation by the child's social background and age.

IV 2 social background				IV 1 age		
Lexical items	Disadvantaged	Advantaged	χ^2^(1)	8 years	14 years	χ^2^(1)
Incest	53	56	0.20	55	54	0.17
Fondling	37	36	0.53	43	30	1.67
Discomfort	19	42	7.19*	32	29	0.04
Strange	21	23	0.16	22	22	0.88
Question	18	20	0.01	17	21	0.92
Abuse	25	15	4.40*	26	14	2.97
Consent	17	19	0.00	12	24	5.47*
Rape	21	11	4.95*	23	9	5.62*
Hug	7	19	4.45*	12	14	0.40
Danger	13	11	0.55	10	14	1.12
Trust	15	9	2.52	15	9	1.11
Protect	10	12	0.18	10	12	0.41
Fear	10	11	0.01	9	8	0.76
Listen	12	9	0.95	15	6	3.33
Family	8	14	1.03	4	18	10.77**
Father	7	12	0.80	12	7	0.98
Aggression	12	4	5.32*	8	8	0.03
Unease	2	14	6.54*	8	8	0.03
Ambiguous	6	9	0.29	6	9	0.92
Anxiety	8	7	0.26	7	8	0.19
Communication	5	7	0.13	4	8	1.06
Intimacy	6	5	0.27	5	6	0.20
Vigilance	7	4	0.68	3	8	1.87
Habit	3	6	1.03	3	8	1.87
Inappropriate	3	7	0.58	6	4	0.03
Distress	6	4	0.27	6	4	0.03

*Note*: The lexical items reported here are ranked by frequency. Only words with a minimum occurrence of 10 were included in the analysis.

Abbreviations: DV, dependent variable; IV, independent variable.

* *p* < .05.

** *p* < .01.

The multivariate GLM revealed significant main effects of both age and social background on participants' judgements, whereas no significant Age × Social Background interactions were observed (see Table 3). Social background accounted for the largest and most consistent effects across the dependent variables.

**TABLE 3:** Results of the general linear model for the effects of age and social background on participants' judgements.

DV	IV 1 age			IV 2 social background		
	8 years *M* (SD)	14 years *M* (SD)	*F*	Disadvantaged *M* (SD)	Advantaged *M* (SD)	*F*
Minimisation of the situation's severity	2.14 (0.06)	2,01 (0.06)	2.49	1.93 (0.06)	2.22 (0.06)	11.49***
Non‐recognition of the abusive nature of the situation and its impact	2.95 (0.09)	3.03 (0.09)	0.33	2.92 (0.09)	3.06 (0.09)	1.35
Exoneration of the father	3.03 (0.06)	3.24 (0.07)	0.10	3.08 (0.07)	3.36 (0.06)	8.70**
Sexualisation of the father's intentions	4.21 (0.08)	4.08 (0.08)	1.23	4.15 (0.08)	4.14 (0.08)	0.01
Blame and disqualification of the minor	2.58 (0.07)	2.43 (0.08)	2.04	2.32 (0.08)	2.70 (0.07)	12.57***
Attribution of silence and inaction to the minor	4.09 (0.10)	4.37 (0.10)	4.05*	4.21 (0.10)	4.25 (0.09)	0.07
Institutional inaction	3.10 (0.08)	3.23 (0.08)	1.25	2.94 (0.08)	3.38 (0.08)	14.91***
Family mediation and internal investigation	4.10 (0.08)	3,84 (0.08)	5.08*	3.93 (0.08)	4.01 (0.08)	0.52
Perceived negative consequences of reporting	3.35 (0.10)	3.29 (0.11)	0.10	3.40 (0.11)	3.35 (0.10)	0.11

Abbreviations: DV, dependent variable; IV, independent variable; *F*, statistic F; *M*, mean; SD, standard deviation.

* *p* < .05.

** *p* < .01.

*** *p* < .001.

Regarding age, two dependent variables displayed small but significant effects. The attribution of silence and inaction to the minor, *F*(7.43) = 4.05, *p* < .045, *ηp* ^*2*^ = .01, was higher for the 14 year old minor (*M* = 4.37, SD = 0.10) than for the 8 year old (*M* = 4.09, SD = 0.10). For family mediation and internal investigation, *F*(6.57) = 5.08, *p* < .025, *ηp* ^*2*^ = .01, these measures were perceived as more appropriate when the minor was 8 years old (*M* = 4.10, SD = 0.08) rather than 14 years old (*M* = 3.84, SD = 0.08).

Regarding social background, substantial effects emerged. Minimisation of the situation's severity, *F*(7.65) = 11.49, *p* < .001, *ηp* ^*2*^ = .03, was stronger when the minor came from an advantaged background (*M* = 2.22, SD = 0.06) compared to a disadvantaged background (*M* = 1.93, SD = 0.06). Father exoneration, *F*(7.28) = 8.70, *p* < .003, *ηp* ^*2*^ = .02, was also higher for fathers from advantaged backgrounds (*M* = 3.36, SD = 0.07) than disadvantaged ones (*M* = 3.08, SD = 0.07). Minor blame and disqualification, *F*(13.09) = 12.57, *p* < .001, *ηp* ^*2*^ = .03, was stronger when the minor came from an advantaged background (*M* = 2.70, SD = 0.07) rather than a disadvantaged one (*M* = 2.32, SD = 0.08). Finally, institutional inaction, *F*(18.57) = 14.91, *p* < .001, *ηp* ^*2*^ = .04, was seen as more appropriate when the family was from an advantaged background (*M* = 3.38, SD = 0.08) versus a disadvantaged one (*M* = 2.94, SD = 0.08). No significant effects were found for the remaining dependent variables.

## DISCUSSION

The findings will be discussed at two complementary levels. First, we examine the empirical findings by triangulating the qualitative and quantitative results across the two studies, with particular attention to the socio‐representational logic underlying professional judgements and intended practices. Second, we discuss the theoretical and methodological contributions of this research, focusing on the value of contextualised operationalisation for investigating how social representations are activated in situations of CSA.

### Socio‐representational logic underlying professional judgements: Triangulation of qualitative and quantitative findings

By articulating three complementary levels of qualitative and quantitative analysis – the representational content identified through interviews in Study 1, the spontaneous semantic associations elicited in Study 2, and the explicit judgements regarding responsibility and professional responses – this research makes it possible to examine the socio‐representational logic that shapes professional judgements and practices regarding intrafamilial CSA. This triangulation highlights how socially shared meanings provide interpretative frameworks through ambiguous situations.

The findings across the two studies suggest that social background not only influences the interpretative process, but also shapes the explicit judgements and decisions involved in practice itself. In Study 1, participants' accounts revealed that ambiguous CSA situations were interpreted through socio‐representational systems of childhood and adolescence, family relationships, parental intentions, children's credibility and the presumption of parental innocence. In Study 2, these representations appeared to be differently activated depending on whether the family was portrayed as socially advantaged or disadvantaged. When the family was described as socially advantaged, participants spontaneously drew on semantic associations related to discomfort and physical closeness. These associations suggest that the situation remained framed within a register of ambiguity and relational interpretation, where the father's behaviour could be understood as inappropriate or problematic without being immediately categorised as violent. Conversely, disadvantaged families elicited associations more directly related to aggression and rape, indicating a more immediate recognition and categorisation of the situation as abusive. This difference in semantic framing was reflected in explicit judgements. When the family was portrayed as advantaged, participants were more likely to minimise the severity of the situation, reduce the father's responsibility, attribute greater responsibility to the minor, and consider institutional inaction more appropriate. Doing nothing in the absence of ‘objective facts’, encouraging internal resolution without alerting authorities, or abandoning reporting were responses more tolerated when the family was described as advantaged. Conversely, disadvantaged families were associated with stronger perceptions of risk, urgency and greater expectations of intervention. Taken as a whole, these results suggest that social background operates as an interpretative framework that influences both the spontaneous construction of meaning and the subsequent judgements and professional actions. These findings extend previous research showing that allegations of sexual abuse involving socially advantaged families are less readily considered credible because these families are associated with competence, respectability, and trustworthiness (Gibbs et al., 2024; Najdowski & Bernstein, 2018). However, the present study contributes to this literature by showing that the influence of social background does not operate only through the recognition or rejection of abuse allegations. Rather, even when participants identify the situation as involving sexual violence, social background continues to shape how responsibility is distributed and how intervention is evaluated. In the case of advantaged families, abusive behaviour may remain embedded within competing interpretations involving family bonds, parental intentions or relational ambiguity. In contrast, disadvantaged families appear to be more readily incorporated into representations of risk, dysfunction, and violence, facilitating the categorisation of the situation as requiring institutional intervention. This distinction is important because it shifts the understanding of social inequalities in CSA responses from a difference in recognition of violence to a broader process through which social characteristics influence the meaning attributed to the same facts.

An important finding concerns the tendency to reduce the father's responsibility and attribute responsibility to the minor when the family was socially advantaged. This pattern suggests that social privilege may operate through mechanisms for the redistribution of responsibility. When the father belongs to a socially valued group, representations associated with respectable, competent, and protective parenthood may conflict with the possibility of sexual abuse. In such contexts, maintaining coherence between the social identity attributed to the family and the existence of violence may involve shifting interpretative attention towards alternative explanations. The results therefore suggest a displacement process: while the situation may be recognised as problematic, the father is less clearly positioned as responsible, whereas the minor is more readily considered as having contributed to the situation. Such a process illustrates how socio‐representational mechanisms may operate not through the denial of facts, but through their reinterpretation within existing social hierarchies (Jost & Banaji, 1994). This finding resonates with research showing that socially advantaged groups benefit from a presumption of credibility and respectability, whereas disadvantaged groups are more frequently associated with suspicion of adverse situations (Gibbs et al., 2024; Najdowski & Bernstein, 2018). Applied to intrafamilial CSA, this suggests that social inequalities may influence not only whether victims are believed, but also how responsibility is attributed once violence is acknowledged. These findings can be interpreted through Bourdieu's (1998) concept of symbolic violence, which refers to the process through which social hierarchies become incorporated into perception and judgement. Social advantages operate not only through material resources but also through symbolic legitimacy, shaping what appears plausible, credible and worthy of institutional intervention. From this perspective, advantaged families benefit from a form of symbolic protection that may make the occurrence of violence within these environments more difficult to reconcile with dominant representations of respectable family life. Abuse may therefore be reframed as ambiguity, misunderstanding or inappropriate but non‐violent behaviour. Conversely, disadvantaged families are more readily associated with representations of dysfunction, danger or risk, which facilitate the interpretation of the same situation as requiring intervention.

Although less pronounced than social background effects, age also influenced the meaning attributed to the situation. Study 1, revealed how social representations of childhood and adolescence, including the notions of autonomy and vulnerability associated with these life stages, shaped participants' interpretations of the situation and whether they recognized it as violence. Study 2 showed that these representations were differently activated depending on whether the minor was described as 8 or 14 years old. The adolescent victim elicited associations related to consent and family relationships, reflecting a representation of adolescence as a period associated with greater agency and responsibility (Brey et al., 2022; Gibbs et al., 2024). This interpretative framework was consistent with judgements attributing greater silence and inaction to the 14 year old minor. Conversely, the younger child was more readily associated with rape and vulnerability, supporting stronger expectations of protection and external intervention.

In intrafamilial CSA response contexts, ambiguity is embedded in complex affective, relational, and socio‐symbolic dynamics that create room for beliefs and interpretive schemas that can minimise and legitimise violence while affecting the quality of care and protection provided. This socio‐representational logic is present in practice and indicates a risk of unequal care depending on the victim's social characteristics. Taken as a whole, these findings suggest that ambiguity in CSA situations allows socio‐representational logic to shape responses in ways that may legitimise unequal care according to victims' social characteristics. Social hierarchies and the beliefs attached to them shape professional judgements by influencing whose suffering appears credible, whose voice is heard, and which situations are considered worthy of institutional intervention. Social class and age therefore contribute to the construction of meaning in CSA situations, thereby participating in the reproduction of underlying social hierarchies through professional judgements and responses.

### Theoretical and methodological contributions: Identifying socio‐representational logic through contextualised operationalisation

Beyond empirical results, this research offers a methodological contribution by proposing a contextualised and sensitive operationalisation of professional judgements in cases of CSA. Using an ambiguous vignette developed from a qualitative approach represents an important methodological innovation. This choice makes it possible to reveal the interpretative frameworks participants rely on without directly inducing them, while preserving the ambiguity inherent in real‐life disclosures. The scenarised vignette thus provides access to interpretation processes activated by the social envelope (Deconchy, 2002) and institutional context, reflecting the complexity, moral tension, and social regulation involved in responding to the care of intrafamilial CSA.

Theorising ambiguity through the SRT allows us to understand how social factors intervene when interpretation is uncertain (Moscovici, 1976). The effects observed show that variations in judgement linked to social markers do not simply reflect differences in opinion but rather reveal social logic grounded in socially shared norms and beliefs that help manage ambiguity in order to respond to evaluative and decision‐making demands. This social logic operates at different levels of interpretation: the free‐association task reflects spontaneous and implicit representational processes activated immediately by the vignette, whereas questionnaire responses are more explicit and deliberated evaluative judgements. Thus, professional judgements and practices vary according to social markers precisely because the situation is ambiguous. In other words, social factors structure the interpretation of the situation, serving as a frame of reference when faced with moral and factual ambiguity. In the context of the care of CSA, judgements are therefore based on an interpretation process activated by context, in which social norms, stereotypes (e.g., class), institutional expectations, and representations (e.g., of childhood or violence) play a decisive role.

This approach is based on a social psychological perspective emphasising the need to contextualise judgement and practice (Apostolidis & Dany, 2012). The vignette emerges as a heuristically valuable tool for capturing situated judgement processes (Deconchy, 2002). By articulating common sense, social context and cognitive logic (Apostolidis, 2006; Doise, 2011), this approach contributes to an anchored psychosocial methodology capable of jointly addressing the content and the process of representations that guide professional judgement and practice. The specificity of SR lies precisely in this dual approach: exploring the cognitive content of representations, drawn from discursive material, and examining the social dynamics at play in their construction (Jodelet, 2015).

The findings confirm that judgements formulated by future professionals are socially situated and regulated by beliefs (Restivo et al., 2016). These judgements are not simply individual evaluations but part of a system of socially shared meanings rooted in and reinforcing normative and ideological frameworks produced by the social context in which they move (Doise, 2003). Judgements about the victim, the perpetrator and the situation bring into play social relations and socio‐representational logic related to class and age, which guide interpretation and decision‐making (Doise, 2003).

### Limitations and future directions

Several limitations should be noted. First, the sample consisted exclusively of trainees in the health and social care professions, which may limit the generalisability of findings to practicing professionals. Comparing these two populations would clarify whether professional socialisation mitigates, maintains, or reinforces the influence of social markers on judgements. Second, the study does not consider the intersection of other social markers, such as gender, ethnicity or disability status. An intersectional perspective would provide a better understanding of the complexity of social categorisations structuring professional judgements. Third, the observed age effect remains limited. Further work should examine representations of childhood and adolescence and contextual conditions that activate them more strongly. Moreover, several dependent subscales showed relatively low internal consistency. These reliability levels may have reduced the accuracy of some measurements. Finally, the current awareness of CSA may lead to a moral ceiling effect, reducing response variability, particularly in more explicitly normative dimensions.

These findings pave the way for further research on the socio‐representational logic involved in CSA care, in particular, an examination of the extent to which judgements are regulated by adherence to adult and masculine domination ideologies, which structure social relations and influence the legitimisation or disqualification of victims' and perpetrators' voices. Finally, these results highlight the need for reflection on socio‐representational logic in CSA care within health and social training programs. Further analysis of these dynamics appears essential for strengthening professional training, reducing the influence of social markers on judgements and decisions, and, more broadly, meeting a major societal challenge: recognising and protecting minors who are victims of sexual violence.

## AUTHOR CONTRIBUTIONS

**Ophélie Gallardo:** Conceptualization; writing – original draft; methodology; investigation; data curation; software; validation; writing – review and editing; formal analysis. **Thémis Apostolidis:** Validation; conceptualization; supervision. **Solveig Lelaurain:** Conceptualization; writing – review and editing; validation; software; supervision; formal analysis.

## FUNDING INFORMATION

This research was supported by Aix Marseille University and conducted at the Laboratoire de Psychologie Sociale (LPS, EA 849).

## CONFLICTS OF INTEREST STATEMENT

The authors declare no conflicts of interest.

## ETHICAL STATEMENTS

These studies received approval from the Ethics Committee Aix Marseille University; 2024‐05‐23‐01.

## Supporting information


**Appendix S1.** Study 1. Semi‐structured interview guide.

**Appendix S2.** Study 2. Questionnaire.

## Data Availability

The data that support the findings of this study are available in a public repository. https://osf.io/amcdj/overview?view_only=d8b9459733ad4c4c8b7b6d8bfd15e6cc.

## Footnotes

^1^
French National Authority for Health.

^2^
All participant names reported in the excerpts are pseudonyms to preserve anonymity.
