# Supplementary material for: Social markers in the care of intrafamilial child sexual abuse: A socio‐representational approach and its theoretical and methodological implications
Source: Br J Soc Psychol. 2026 Oct 22;65(4):e70145. doi: 10.1111/bjso.70145 (PMC13691282; doi:10.1111/bjso.70145)
Supplement: Supplementary file 1 [file BJSO-65-0-s001.docx]

**Appendix 1.**

**Study 1. Semi-structured interview guide**

**Main questions**

1. How would you describe this situation?
2. What does this situation evoke for you?
3. In your opinion, what happened to Clara?
4. How do you perceive Clara's father? And Clara?
5. How do you imagine the family context in which they live?
6. How do you think the school nurse would respond to what Clara has disclosed?

**Additional prompts (used if these issues were not spontaneously addressed by the participant)**

7. Why do you think Clara chose to disclose this situation to the school nurse?
8. What do you think Clara expects from the school nurse?
9. In your opinion, is this situation an isolated case or something that is likely to occur repeatedly?
10. Do you think this situation could come to an end? If so, how?
11. How would you assess the seriousness of this situation? Why?
12. Do you think this situation could be considered a case of child sexual abuse? Why or why not?
13. What do you think of the vignette? Does it seem realistic to you?
14. If Clara were 8 rather than 15 years old, would your perception of the situation be different? What if her father were unemployed rather than a notary?
