# Supplementary material for: Social markers in the care of intrafamilial child sexual abuse: A socio‐representational approach and its theoretical and methodological implications
Source: Br J Soc Psychol. 2026 Oct 22;65(4):e70145. doi: 10.1111/bjso.70145 (PMC13691282; doi:10.1111/bjso.70145)
Supplement: Supplementary file 2 [file BJSO-65-0-s002.docx]

**Questionnaire**

*The questionnaire was translated from French into English for the purposes of this study.*

**Hello,**

This study is being conducted by a research team from [Laboratory/University].

The study aims to examine the factors that shape impression formation in situations involving the care of minors.

This questionnaire is intended for students in nursing or social work. It takes approximately 20 minutes to complete.

We are interested in your opinions and ask you to answer based on what you genuinely think. There are no right or wrong answers.

Your responses will remain strictly confidential and anonymous and will be used solely for the purposes of this study.

Strict anonymity means that your responses cannot be traced back to you. It also makes it impossible to correct or delete your information after you have completed the study and the data have been recorded.

The data will be processed anonymously and will not be linked to your identity. The anonymization procedure ensures that no individual can be identified, even indirectly.

The data will be stored in an anonymous form for as long as necessary for research purposes by the principal investigator.

They will be protected in accordance with applicable data protection laws, including Regulation (EU) 2016/679 of the European Parliament and of the Council of April 27, 2016, on the protection of individuals with regard to the processing of personal data and on the free movement of such data (General Data Protection Regulation, GDPR).

There are no anticipated risks associated with participating in this study.

You may stop participating at any time without any consequences.

You will be able to access the results of the study once the data have been analyzed and the study has been completed.

By selecting “Yes,” I confirm that I have read and understood the information provided above and freely and voluntarily agree to participate in the research study entitled “C*ollecting Impressions in the Care of Minors*”.

*Yes	No*

I confirm that I am a student in nursing or social work.

*Yes	No*

***Vignettes:*** *Participants were randomly presented with one of four vignettes (Vignette 1, 2, 3, or 4). They were asked to read the scenario before answering the questions that followed.*

1. What are the three words that spontaneously come to mind after reading this situation?

*Word 1	Word2	Word 3*

2. Below is a series of statements concerning the situation described by Clara.
   *Please indicate the extent to which you agree or disagree with each statement by rating it on a scale from 1 (“Strongly disagree”) to 7 (“Strongly agree”).*

**2.1** In my opinion, the situation described by Clara is not serious.

**2.2** In my opinion, the situation described by Clara is too ambiguous to be taken seriously.

**2.3** In my opinion, the situation described by Clara can be explained by her father’s problems.

**2.4** In my opinion, the situation described by Clara is most likely a misunderstanding.

**2.5** In my opinion, the situation described by Clara will most likely have long-term consequences for her.

**2.6** In my opinion, the situation described by Clara is probably a situation of sexual abuse.

### **3.** Below is a series of statements concerning the people involved in the situation described by Clara.

### *Please indicate the extent to which you agree or disagree with each statement by rating it on a scale from 1 (“Strongly disagree”) to 7 (“Strongly agree”).*

**3.1** Clara’s father’s actions are certainly not intended to cause harm.

**3.2** At her age, Clara probably wants people to pay attention to her.

**3.3** Clara’s father did not behave appropriately.

**3.4** Clara should have expressed her discomfort to her father.

**3.5** Clara’s father may be attracted to his daughter’s body.

**3.6** Like many people her age, Clara sometimes distorts reality.

**3.7** Clara’s father experienced a sexual urge.

**3.8** Clara was unable to oppose her father.

**3.9** Clara’s father is like all other fathers.

**3.10** At her age, Clara is telling this story to cause problems for her father.

**3.11** Within Clara’s family, Clara’s father exerts a form of control over her.

**3.12** Clara most likely misinterpreted her father’s actions.

**3.13** Clara’s father is not dangerous.

**3.14** Clara is not under her father’s control.

**3.15** Clara’s father is affectionate.

**3.16** Like others her age, Clara is probably going through a period of seeking attention and attraction.

### **4.** In your opinion, what should the nurse do in response to the situation described by Clara? *For each of the following options, indicate how appropriate you consider it by rating it on a scale from 1 (“Not at all appropriate”) to 7 (“Very appropriate”).*

**4.1** The nurse will reassure Clara by telling her that there is nothing unusual about the situation.

**4.2** The nurse will advise Clara to tell her father to stop.

**4.3** The nurse should verify what Clara has told her.

**4.4** The nurse will maintain professional confidentiality.

**4.5** The nurse will not take any action because there is insufficient objective evidence.

**4.6** The nurse will inform Clara’s father.

**4.7** The nurse will offer to meet with Clara’s parents.

**4.8** The nurse will try to resolve the situation internally, without informing the authorities.

**4.9** The nurse will submit a report to the Cellule de Recueil des Informations Préoccupantes (CRIP) so that social workers can take the necessary measures.

**4.10** The nurse will report the situation to the Public Prosecutor’s Office so that a police investigation can be initiated.

### **5.** *Please indicate the extent to which you agree or disagree with the following statements by rating each one on a scale from 1 (“Strongly disagree”) to 7 (“Strongly agree”).*

**5.1** In my opinion, the nurse will take this situation seriously.

**5.2** Reporting the situation could have negative consequences for Clara.

**5.3** Reporting the situation could have negative consequences for the nurse.

### **6.** Some information about you. Finally, we would like to ask you a few questions about yourself:

**6.1** How do you describe your gender identity?

*I am a woman	I am a man	Other*

**6.2** What is your age?

**6.3** What is your field of study?

*Nursing	Social work	Other*

**6.4** What year of study are you currently in?

*1st year	2nd year	3rd year	Other*

**6.5** Do you know one or more people who experienced sexual abuse during childhood?

- *Yes, among celebrities and/or public figures*
- *Yes, among distant acquaintances*
- *Yes, among my friends*
- *Yes, among my family members*
- *No, I do not know anyone*

**6.6** During your childhood, did an adult ever behave toward you in a sexual manner, including through sexualized attitudes, gestures, or behaviors?

*Yes	No	I don't know	I prefer not to answer*

**6.7** Do you feel that you are well informed about child sexual abuse?

*Yes	No*

**6.8** Have you ever participated in training on child sexual abuse?

*Yes	No*

**6.9** If you have any comments about this study, please enter them below:

**Thank you for your participation.**

For any questions regarding the study, you can contact us at the following email address: [Author 1]

The situation you read about in this study was fictional.

You have just participated in a psychology study examining the psychosocial factors involved in impression formation in situations involving the care of minors.

If, following the study, you feel the need to speak with someone, we have provided below some organizations that offer free, anonymous, safe, and supportive services:

- *CIIVISE: 0 805 802 804*
- *CIDFF: 04 96 11 07 99*
- *Collectif Féministe Contre le Viol: 0 805 802 804*
- *Planning Familial 13: 0 800 08 11 1*
- *Solidarité Femmes 13: 04 91 24 61 50*
